# Dysarthria Subgroups in Talkers with Huntington’s Disease: Comparison of Two Data-Driven Classification Approaches

**DOI:** 10.3390/brainsci12040492

**Published:** 2022-04-13

**Authors:** Daniel Kim, Sarah Diehl, Michael de Riesthal, Kris Tjaden, Stephen M. Wilson, Daniel O. Claassen, Antje S. Mefferd

**Affiliations:** 1 Department of Hearing and Speech Sciences, Vanderbilt University Medical Center, Nashville, TN 37232, USA; sarah.k.diehl@vanderbilt.edu (S.D.); michael.r.de.riesthal@vumc.org (M.d.R.); stephen.m.wilson@vanderbilt.edu (S.M.W.); antje.mefferd@vumc.org (A.S.M.); 2 Department of Communicative Disorders and Sciences, University at Buffalo, Buffalo, NY 14260, USA; tjaden@buffalo.edu; 3 Department of Neurology, Vanderbilt University Medical Center, Nashville, TN 37232, USA; daniel.claassen@vumc.org

**Keywords:** dysarthria, auditory free classification, perceptual speech assessment, perceptual speech characteristics

## Abstract

Although researchers have recognized the need to better account for the heterogeneous perceptual speech characteristics among talkers with the same disease, guidance on how to best establish such dysarthria subgroups is currently lacking. Therefore, we compared subgroup decisions of two data-driven approaches based on a cohort of talkers with Huntington’s disease (HD): (1) a statistical clustering approach (STATCLUSTER) based on perceptual speech characteristic profiles and (2) an auditory free classification approach (FREECLASS) based on listeners’ similarity judgments. We determined the amount of overlap across the two subgrouping decisions and the perceptual speech characteristics driving the subgrouping decisions of each approach. The same speech samples produced by 48 talkers with HD were used for both grouping approaches. The STATCLUSTER approach had been conducted previously. The FREECLASS approach was conducted in the present study. Both approaches yielded four dysarthria subgroups, which overlapped between 50% to 78%. In both grouping approaches, overall bizarreness and speech rate characteristics accounted for the grouping decisions. In addition, voice abnormalities contributed to the grouping decisions in the FREECLASS approach. These findings suggest that apart from overall bizarreness ratings, indexing dysarthria severity, speech rate and voice characteristics may be important features to establish dysarthria subgroups in HD.

## 1. Introduction

Dysarthria is a neurogenic motor speech disorder that impairs speech motor execution due to paralysis, weakness, and/or incoordination [1]. Talkers with dysarthria differ in their perceptual speech characteristics. Although some of these perceptual characteristics can be observed in virtually all talkers with dysarthria (e.g., imprecise consonants), other speech characteristics are unique to only some talkers (e.g., strained-strangled voice). Therefore, a dysarthria classification system is needed to account for this heterogeneity of dysarthria symptoms and to provide a conceptual framework of dysarthria as a neurogenic motor speech disorder.

Within the classic Mayo Clinic dysarthria classification system, some neurological diseases are associated with specific dysarthria types that are further characterized by specific perceptual speech patterns [2,3,4]. Darley and his colleagues, for example, determined that Parkinson’s disease (PD) is associated with hypokinetic dysarthria and is characterized by reduced loudness (hypophonia) and a variety of impaired articulation (e.g., fast/variable rate and short rushes of speech). Similarly, Huntington’s disease (HD) is associated with hyperkinetic dysarthria and characterized by prolonged intervals, a variable or reduced articulation rate, imprecise consonants, and excess intensity variations [1,5]. Similarly, within the Mayo Clinic dysarthria classification framework, a perceptual speech pattern can be characteristic of a specific disease (e.g., myasthenia gravis) and help facilitate its diagnosis or be suggestive of specific neuropathology (e.g., bilateral upper motor neuron lesions) [1]. However, recent perceptual studies have also shown that perceptual speech patterns of individuals who share a disease that is conventionally associated with a specific dysarthria type (e.g., HD → hyperkinetic dysarthria) can vary greatly and may even resemble perceptual speech patterns of dysarthria types that are typically associated with other neurologic conditions with unrelated underlying neuropathologies (e.g., cerebellar ataxia) [6]. Therefore, it is not surprising that clinicians have reported difficulties with the differential diagnosis of dysarthria, even when they were trained to use the Mayo Clinic dysarthria classification system [7]. Given these challenges, the question arises of how best to group talkers with dysarthria to adequately account for the heterogeneous perceptual speech characteristics that can be found within disease types that are typically associated with one specific dysarthria type.

For about a decade, dysarthria subgroup approaches have been sought out to address the heterogeneity of dysarthria manifestation within disease types [8,9]. These approaches sought to establish subgroups of talkers with similar perceptual speech patterns using a “bottom-up” approach; that is, acoustic or perceptual speech characteristics rather than the disease type formed the basis for their grouping decisions. Diehl and colleagues (2019) [10] submitted the perceptual speech profiles of 48 talkers with HD to an unsupervised k-means cluster analysis (hereafter referred to as the STATCLUSTER approach). The perceptual speech profiles of these talkers with HD were established by six judges who were trained to assess perceptual speech characteristics based on the Mayo Clinic dysarthria rating scale [2]. The scale consists of 38 dimensions (i.e., perceptual speech characteristics) that are rated using a 7-point scale (1 = normal, 7 = severely deviant). Four dysarthria subgroups were identified. These subgroups predominantly differed in ratings of perceived speech rate and overall perceived bizarreness and intelligibility (i.e., slow rate/mild-dysarthria, slow rate/moderate-dysarthria, adequate rate/mild-dysarthria, fast rate/mild-dysarthria).

Presumably, the STATCLUSTER approach, when based on perceptual speech profiles, will create subgroups of talkers with dysarthria whose deviant speech patterns are perceptually similar due to overlapping perceptual speech profiles. However, this notion hinges on the assumption that there is a linear association between the severity rating of the deviant perceptual speech characteristics and their perceptual saliency as one aspect of the overall perceptual speech pattern. Indeed, Darley and colleagues reported that some dimensions (i.e., perceptual speech characteristics) of the classic Mayo Clinic dysarthria rating scale frequently received greater mean scale values than others and speculated that these dimensions might be “more interesting” to listeners [2]. Along these lines, Zyski and Weisiger (1987) [11] suggested that perceptual speech characteristics with greater mean scale values may be “more salient” to listeners and may predominantly account for the perceptual-based differentiation of dysarthria types.

However, it is also conceivable that the association between the rating of a deviant perceptual speech characteristic and its perceptual saliency is nonlinear. That is, the presence of a deviant perceptual speech characteristic, even when barely detectable (e.g., a strained-strangled voice quality), may stand out and distinguish some talkers with dysarthria from their peers. Such perceptual saliency may even occur when the severity rating of the deviant perceptual speech characteristic does not differ from those of other deviant perceptual speech characteristics that are shared by most talkers within the cohort. Stated differently, the interval scale used for the severity rating of each perceptual speech characteristic in the Mayo classification system may not always accurately represent the perceptual magnitude between each point on the interval scale [12,13].

The auditory free classification task (from here on referred to as FREECLASS) is an approach that can also be used to create talker subgroups based on their perceptual speech patterns [14]. However, in contrast to the STATCLUSTER approach where perceptual speech profiles are subjected to cluster analysis, the talker groups that result from a FREECLASS approach are solely driven by the saliency of perceptual speech characteristics [6,14,15,16]. Specifically, during a FREECLASS approach, listeners are instructed to establish subgroups of talkers with dysarthria who share similar perceptual speech patterns. However, unlike the STATCLUSTER approach, no specific list of perceptual speech characteristics or severity rating scale is provided to the listeners. Instead, FREECLASS requires that listeners establish their own sorting rules based on the various speech samples.

Clopper and colleagues (2004) [17] were some of the earliest researchers to employ the auditory FREECLASS approach using speech stimuli. They demonstrated that listeners could make similarity judgments of talkers’ regional dialects. They further examined the extent to which the findings of the free classification task overlapped with those of a forced-choice categorization task and discovered similar patterns of findings across the approaches. Although the findings of Clopper and colleagues (2004) [17] suggest a close link between perceptual ratings and saliency of perceptual speech characteristics for regional dialects, it is unknown if such a relationship also exists for dysarthria.

Findings in the study by Diehl and colleagues (2019) [10] revealed that subgroups based on the STATCLUSTER approach differed predominantly in their speech rate characteristics. It is possible that speech rate characteristics are most salient for listeners. However, it is also possible that speech rate characteristics predominantly index dysarthria severity. This notion is supported by the fact that bizarreness and speech intelligibility ratings moderately covaried with ratings of speech rate abnormalities, and also significantly differed across subgroups.

In sum, although the heterogeneous manifestation of dysarthria symptoms within disease types (e.g., PD, HD) has long been acknowledged (e.g., [6,10,18,19]), guidance on how to best establish dysarthria subgroups within disease types is still lacking. Therefore, the current study compared grouping decisions derived from a STATCLUSTER approach with those derived from a FREECLASS approach, with both approaches using the same cohort of talkers with HD. Specifically, we sought to determine a) the extent to which these two approaches overlap in their grouping decisions, and b) which perceptual speech characteristics significantly contributed to grouping decisions for each approach.

The findings of this study will provide insights into how different grouping approaches may or may not impact grouping decisions and which specific perceptual speech characteristics can be used to establish dysarthria subgroups for HD. Such knowledge will advance the design of future research studies on dysarthria in HD; for example, research efforts that aim to elucidate pathophysiologic factors that underlie various dysarthria subgroups. Findings will also offer improved study designs for investigations on the clinical management of dysarthria in these talkers.

## 2. Materials and Methods

### 2.1. Participants (Listeners)

The study was approved by the IRB committee of Vanderbilt University Medical Center. Seventeen students (two males and 15 females; mean age = 25.2 years, SD = 3.36 years) enrolled in the Vanderbilt University Graduate Program in Hearing and Speech Sciences participated as listeners. All students successfully completed a graduate-level motor speech disorders course and passed a standard pure-tone hearing screening (500, 1000, 2000, and 4000 Hz at 25 dB HL in both ears). The criteria to include students who have completed graduate-level motor speech disorders paralleled the approach taken by Lansford and colleagues [6] and ensured that listeners were familiar with deviant perceptual speech characteristics of talkers with dysarthria. All listeners were recruited using departmental emails and consented to the study. In addition, they were compensated for their time.

### 2.2. Talkers and Stimuli

All speech audio recordings were accessed retrospectively from Diehl et al. (2019) [10]. The auditory stimuli consisted of speech samples produced by 48 talkers (16 males, 32 females; mean age = 51.5 years, SD = 13.5 years) diagnosed with HD by a board-certified neurologist and confirmed via genetic testing. All speakers spoke English as their native language and did not have a diagnosis of other neurologic conditions. All talkers with HD presented with dysarthria as determined through consensus ratings of three certified speech-language pathologists [10]. Sentence intelligibility test scores (SIT) [20] ranged between 80% and 100%.

In addition to reading the SIT sentences, every talker with HD completed a standardized speech assessment using the Rainbow Passage [21]. Both speech samples were recorded using a Tascam digital recorder ([Santa Fe Springs, CA, USA] DR-100MKII) and a lapel microphone (Audio-Technica [Tokyo, Japan] AT899) with a microphone-to-mouth distance of approximately 6 inches. Each talker’s recording of the Rainbow Passage was edited to include the first three sentences using TF32 [22]. The entire reading passage could not be used for the purpose of this study, considering its duration and the number of talkers included in this study. However, the first three sentences of the Rainbow passage were deemed to adequately represent the perceptual speech characteristics of each talker [23,24]. Sample duration ranged from 8 to 38 s, with a mean duration of 16.3 s.

**Figure 1:**
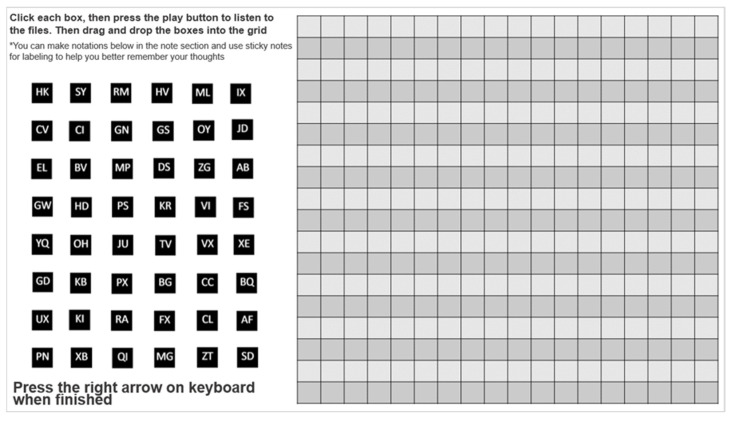
Auditory free classification approach (FREECLASS) paradigm. A listener can play and listen to the speech samples by clicking each box. A listener can then drag and drop the boxes into the grid. At least one side of one box must touch at least one side of another box on the grid for the boxes to be considered members of the same subgroup. * Additional instruction to allow listeners to make notations and comments was provided because the task could potentially pose a high demand for working memory.

The trimmed speech samples were each linked to 48 identical solid gray squares with randomly assigned two-letter identifiers for better retention during the experiment (as in [6]). All speaker stimuli were embedded in a single PowerPoint slide next to a 17 × 18 grid (Figure 1). The slide with embedded speaker stimuli was presented to each listener with instructions.

### 2.3. Rating Each Talker’s Perceptual Speech Characteristics

**Figure 2:**
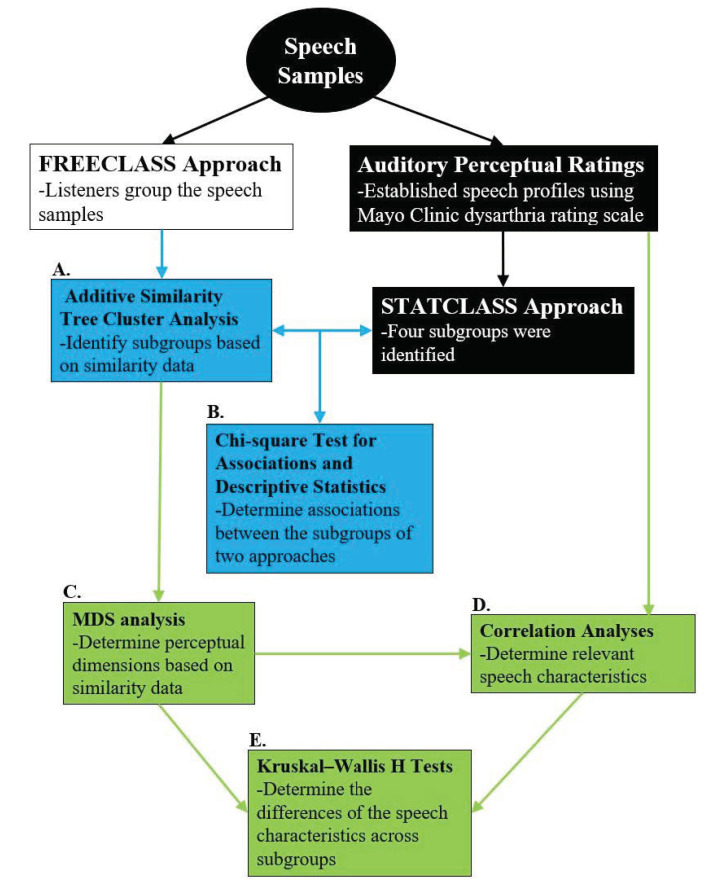
A flowchart depicting the summary of study design procedures. Black boxes indicate data and processes that have been previously collected and completed in Diehl et al., 2019 [10]. Blue boxes indicate methods conducted to determine the overlap between the two group approaches. Finally, green boxes indicate study procedures to determine contributing speech characteristics for the findings of the FREECLASS approach. Please note that the same perceptual speech profiles based on the Mayo Clinic dysarthria rating scale were used in both approaches. (**A**) Additive similarity tree cluster analysis; (**B**) chi-square test for associations and descriptive statistics; (**C**) multidimensional scaling (MDS) analysis; (**D**) correlation analyses; (**E**) Kruskal–Wallis H tests.

In the previous study by Diehl et al. (2019) [10], the perceptual ratings based on the Mayo Clinic rating scale were used as input variables for a STATCLUSTER approach. The same perceptual ratings were utilized in the current study after the dysarthria subgroups were established using the FREECLASS approach to determine the perceptual speech characteristics accounting for subgroup decisions (Figure 2). A brief description of the perceptual ratings completed previously by Diehl and colleagues is provided in the next paragraph.

The audio recordings of the full Rainbow passage of the 48 talkers with HD were rated by six trained raters. These raters were second-year graduate students in speech-language pathology and had completed a 16-week motor speech disorders course. They also received a 2-h training module, during which they were familiarized with the Mayo Clinic dysarthria rating scale and its 38 perceptual speech characteristics [2]. The severity of impairment for each perceptual speech characteristic was rated based on a 7-point scale that ranged from 1 (normal) to 7 (severely impaired). The 38 items belong to one of seven categories: pitch characteristics, loudness, vocal quality, respiration, prosody, articulation, and general perceptual impression of speech. Interrater and intrarater agreements of the perceptual judgments were inspected and deemed adequate (see [10]).

### 2.4. The Auditory Free Classification Task

Each listener completed an auditory free classification task in the Speech Kinematics and Acoustics Lab at Vanderbilt University Medical Center. The listeners were seated in front of a computer equipped with professional grade over-ear headphones (Audio-Technica ATH-M50X). At the start of the study, a sample audio recording of a neurologically healthy talker was presented, who was recorded with the same audio recording setup as the talkers with HD. The listeners were asked to adjust the volume of their headphones to a comfortable loudness level.

Next, the listeners were informed that they would be presented with speech samples of persons with dysarthria; however, the listeners were blinded to the underlying etiology of HD. The listeners were then asked to complete the auditory free classification task using the speech samples of the 48 speakers with HD, which were embedded in a PowerPoint slide next to a grid (see Figure 1). Specifically, the listeners were asked to group the 48 speech samples by clicking, dragging, and dropping each of them into one of the grid cells based on how similar they sounded to the listener. The listeners were instructed to ensure that a stimulus touched at least one side of another stimulus on the grid when the listener wanted to indicate that the two samples belonged to the same group. No limitations were imposed with regards to completion time, number of groups, and number of times the listener could play a speech sample. Because the task could potentially pose a high demand for working memory [6], each listener was allowed to make notations and comments using the note section in the PowerPoint slide. Listeners also were provided with paper and pen as an alternative to the note sections.

### 2.5. Data Analysis

First, the number of the identified subgroups of talkers with HD was recorded for each listener. The mean, median, and range of talker subgroups with HD were also calculated across all listeners to gain a general understanding of the listeners’ performance on the auditory free classification task. To determine which talkers were most frequently grouped together, each listener’s groupings of talkers with HD were transferred into an excel spreadsheet. Next, a macro function established a similarity matrix by assigning a value of 1 for the talker pairs that were grouped together. A value of 0 was assigned for talker pairs that were not grouped together. The resulting 17 similarity matrices were combined to produce a single similarity matrix. Finally, the matrix was submitted to an additive similarity tree cluster analysis [25] using GTREE (procedure A in Figure 2), a Pascal program for fitting additive trees (see [6]). This additive similarity tree cluster analysis was used to identify the subgroups of talkers with HD with perceptually similar speech characteristics, as determined by the listeners during the FREECLASS approach. To determine the extent of overlap between the FREECLASS approach and the STATSCLUSTER approach, the strength of association between the subgroups of talkers with HD identified in the current study and the subgroups of those identified in the study by Diehl and others (2019) [10] was examined using a chi-square test for association (procedure B in Figure 2).

Several steps were taken to determine the perceptual speech characteristics accounting for the subgroup decisions in the FREECLASS approach. First, as in Lansford et al. (2014) [6], the similarity matrix that combined all listeners’ responses from the FREECLASS approach was submitted to PROXCAL Multidimensional Scaling (MDS) analysis using SPSS. The resulting dimensions corresponding to similarity data were then plotted on two-dimensional spaces for visual interpretations of each identified perceptual dimension (procedure C in Figure 2). Then, correlation analyses were performed between the dimensions resulting from MDS and the ratings of perceptual speech characteristics of each talker with HD (procedure D in Figure 2). It should be noted that the sex of each talker was also coded as a variable and submitted to this correlational analysis in addition to the perceptual ratings due to the possibility that listeners could group talkers with HD merely based on the talker’s sex. Finally, to determine the perceptual speech characteristics that significantly differed across subgroups, Kruskal–Wallis H tests were conducted for each speech characteristic that revealed at least a moderate correlation with each dimension (r ≥ 0.4) identified in the MDS analysis (procedure E in Figure 2). If the Kruskal–Wallis H test results were significant, Dunn’s post-hoc tests adjusted with Bonferroni correction were performed to identify the specific between-group patterns. The analysis of sex effects was conducted using Fisher’s exact test because of the binary nature of this variable.

## 3. Results

### 3.1. Determining Numbers of Dysarthria Subgroups among Talkers with HD

**Figure 3:**
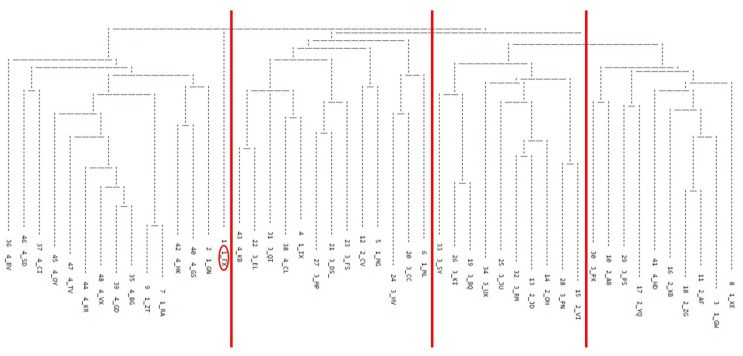
A dendrogram depicting the results of an additive similarity tree cluster analysis. The red lines indicate the boundaries made in defining the identified subgroups of the study. Participant FX, circled in red, did not belong to any of the identified four groups and was excluded from the analyses.

On average, listeners identified 8.5 groups of talkers with similar perceptual speech patterns (SD = 3.0, median: 9, range: 4–13). Each group included an average of 5.6 talkers with HD (SD = 3.9, range: 1–21). The results of the additive similarity tree cluster analysis [25] are presented in Figure 3. The dendrogram represents talkers grouped most frequently together by listeners using the FREECLASS approach. As in Lansford et al. (2014) [6], the dendrogram was visually inspected to determine the number of dysarthria subgroups among talkers with HD. Four dysarthria subgroups were identified. The number of talkers within each subgroup ranged from 10 to 14 (median 11.5). One talker (FX) did not belong to any of the four subgroups and was excluded from further analysis.

### 3.2. Extent of Overlap between the FREECLASS and the STATSCLUSTER Approach

**Figure 4:**
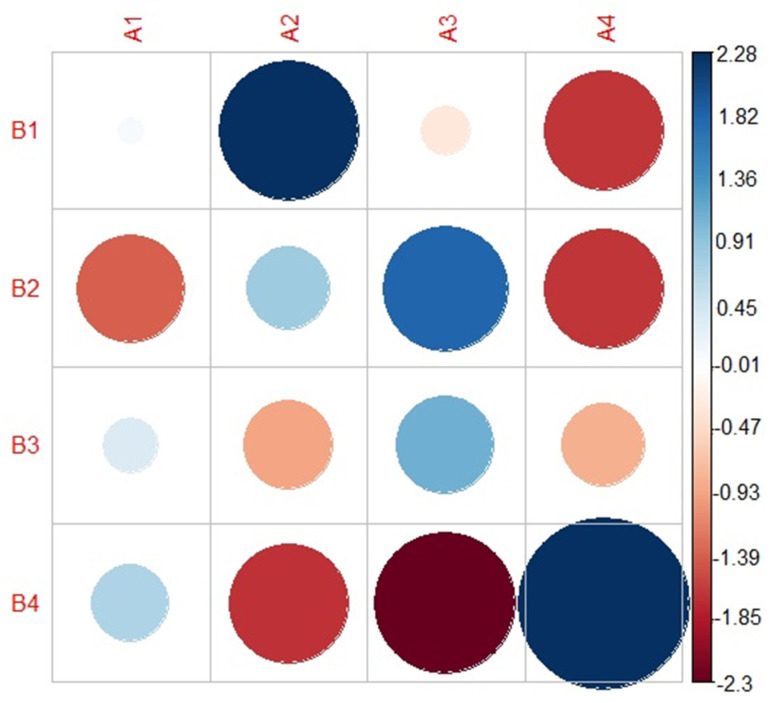
A correlation plot describing the results of the chi-square test for associations. The blue circles indicate the degree of positive associations between the subgroups of the FREECLASS approach and the subgroups of the STATCLUSTER approach. The red circles indicate the degree of negative associations.

A chi-square test for association (see procedure B in Figure 2) revealed a significant positive association between the four subgroups identified using the FREECLASS approach and the four subgroups identified using the STATCLUSTER approach, χ^2^ (9) = 38.38, *p* < 0.01. The descriptive comparisons between the compositions of the subgroups from the two approaches are provided in Table 1, and a corresponding correlation plot can be found in Figure 4. Diehl and colleagues (2019) [10] identified four subgroups of talkers with HD, which predominantly differed in perceived rate and severity (i.e., slow rate/moderate dysarthria, slow rate/mild dysarthria, normal rate/mild dysarthria). Subgroup 1 of the FREECLASS approach overlapped 50% (5 out of 10 talkers) with the previously identified subgroup 2 of the STATCLUSTER approach (labeled as slow rate/moderate dysarthria). Subgroup 2 of the FREECLASS approach overlapped 70% (7 out of 10 talkers) with the previously identified STATCLUSTER subgroup 3 (labeled as slow rate/mild dysarthria). Subgroup 3 of the FREECLASS approach overlapped 53.8% (7 out of 13 talkers) with the previously identified STATCLUSTER subgroup 3 (also labeled as slow rate/mild dysarthria). Finally, subgroup 4 of the FREECLASS approach overlapped 78.6% (11 out 14 talkers) with the previously identified STATCLUSTER subgroup 4 (labeled as adequate rate/mild dysarthria). It is also important to note that subgroup 4 of the FREECLASS approach only contained talkers from subgroups 1 and 4 of the STATCLUSTER approach (labeled as fast rate/mild dysarthria or adequate rate/mild dysarthria, respectively). Additional details on the compositions of the subgroups identified using the FREECLASS approach relative to the subgroups identified using the STATCLUSTER approach are provided in Table 1.

### 3.3. Determining Distinct Differences between Dysarthria Subgroups of Talkers with HD

**Figure 5:**
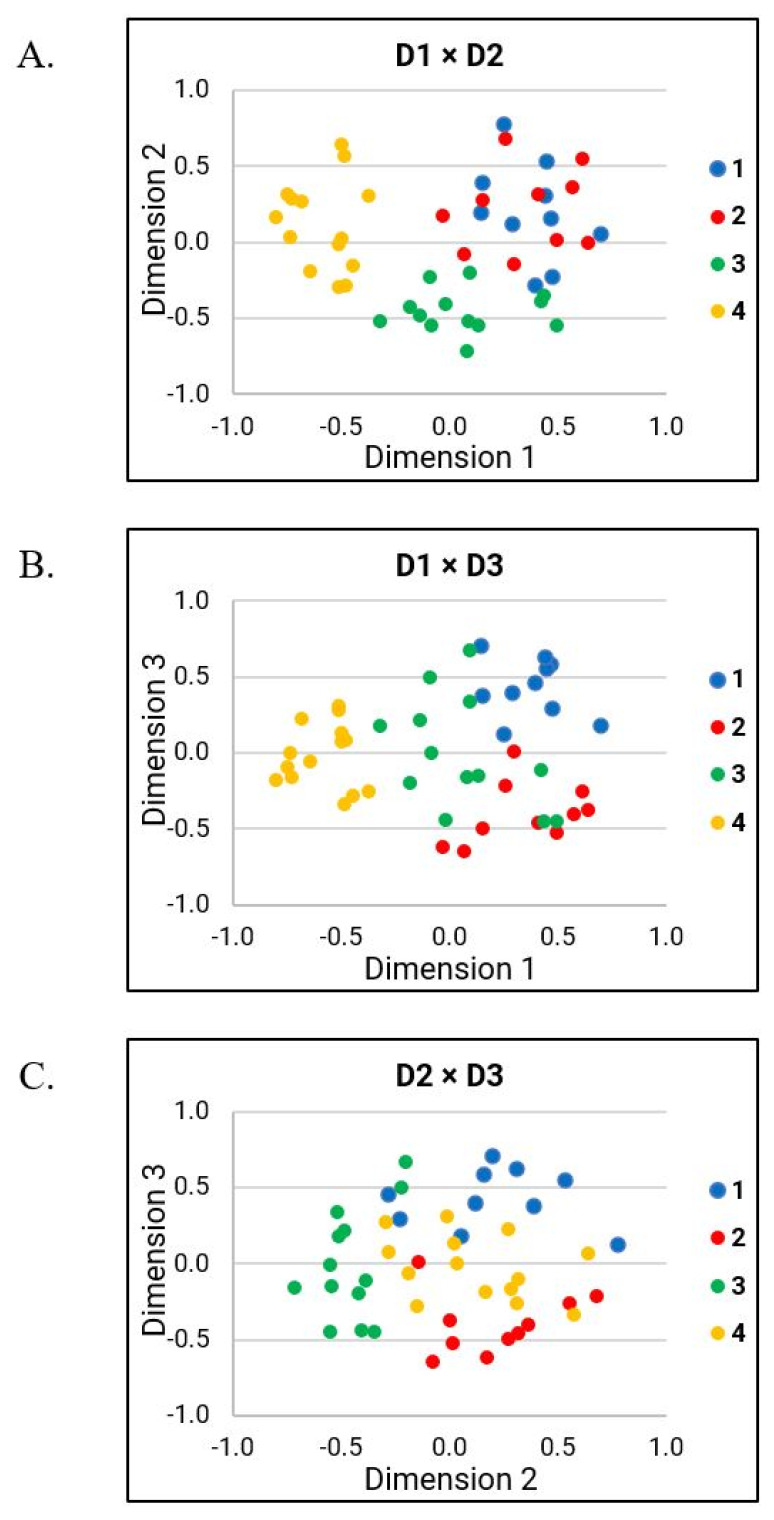
Perceptual similarity data plotted in the common spaces based on the dimensions revealed from MDS analysis. (**A**) A perceptual space defined by Dimension 1 (D1) and Dimension 2 (D2); (**B**) a perceptual space defined by D1 and Dimension 3 (D3); (**C**) a perceptual space defined by D2 and D3.

Multidimensional scaling analysis of the similarity data revealed a three-dimensional model with the normalized raw stress value of 0.035 (dispersion accounted for = 0.964; Stress 1 = 0.189). The three-dimensional model was chosen because it produced the optimal normalized raw stress value. The similarity data of the talkers with HD were then plotted in the common spaces based on the revealed dimensions (see Figure 5). Figure 5A shows the similarity data of talkers using Dimension 1 (D1) and Dimension 2 (D2). Considering D1, subgroup 4 was clearly differentiated from the other three subgroups. Although subgroup 3 also stood out in D1, it overlapped to a greater extent with the other subgroups than subgroup 4. In D2, however, subgroup 3 was the only subgroup that could be clearly distinguished from the other subgroups. In Figure 5B,C, Dimension 3 (D3) was plotted against D1 and D2. As can be seen in these two figures, subgroup 1 and subgroup 2 were clearly differentiated from each other in D3, while overlapping in D1 and D2.

To determine the extent to which each variable (i.e., perceptual rating of each speech characteristic, talker’s sex) contributed to D1, D2, and D3, correlational analyses and nonparametric between-group tests were conducted (see also [6]). Table 2 reports the findings for the perceptual speech characteristics with significant moderate correlations (r > 0.4) and significant between-group effects (*p* < 0.05) based on the Kruskal–Wallis H test or a Fisher’s exact test.

#### 3.3.1. Dimension 1

D1 was associated most strongly with the perceptual ratings of overall bizarreness (r = 0.835) and perceived speech rate (r = −0.71). D1 also revealed significant moderate correlations with several prosodic perceptual speech characteristics (i.e., excess and equal stress, intervals prolonged, inappropriate silences). In addition, D1 showed significant moderate correlations with perceptual ratings of overall intelligibility, as well as several perceptual speech characteristics associated with articulation (i.e., imprecise consonants, irregular articulatory breakdown, phonemes prolonged, vowels distorted), loudness (i.e., excess loudness variation, alternating loudness), and pitch (i.e., monopitch).

**Figure 6:**
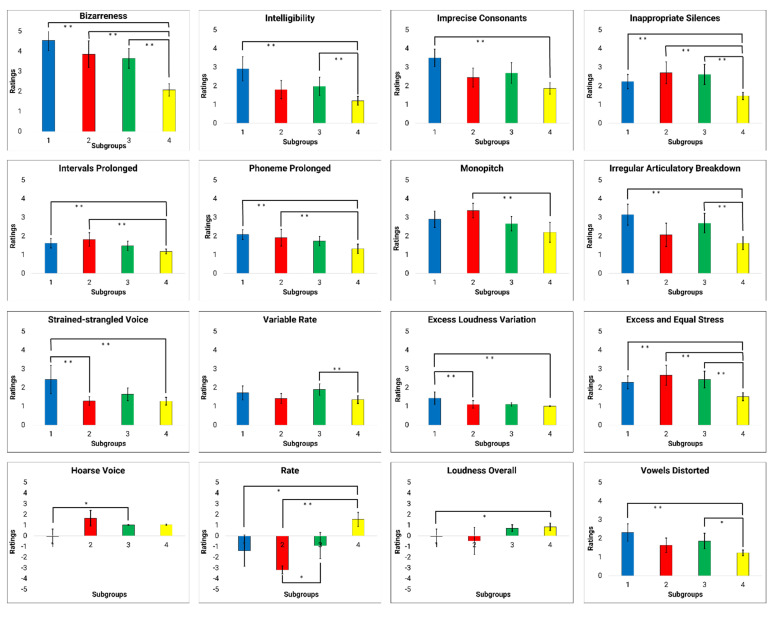
Subgroup means (95% confidence interval) for the ratings of perceptual measures. ** *p* < 0.05; * *p* ≤ 0.084.

As shown in Table 3, Kruskal–Wallis H tests revealed significant between-group effects across the identified subgroups from the FREECLASS approach for all of the perceptual speech characteristics that were at least moderately correlated (r > 0.4) with D1 (*p* < 0.05), except for alternating loudness (*p* = 0.07). Table 4 and Figure 6 display the subsequent results of Dunn’s post-hoc test (adjusted *p*-values according to Bonferroni corrections) for all variables with significant Kruskal–Wallis H test results, and moderately correlated with D1. To start with, subgroup 4 had significantly lower ratings of bizarreness, excess and equal stress, and inappropriate silences relative to the ratings of all other subgroups (*p* < 0.03). Subgroup 4 also had significantly lower ratings of intervals prolonged and phonemes prolonged relative to subgroups 1 and 2 (*p* < 0.05). In addition, between-group comparisons for the ratings of intelligibility and irregular articulatory breakdown showed that subgroup 4 had significantly lower ratings than subgroups 1 and 3 (*p* < 0.04). Finally, subgroup 4 had significantly lower ratings of perceived speech rate and monopitch than subgroup 2 (*p* < 0.01), and had significantly lower ratings of imprecise consonants, vowels distorted, and excess loudness variation than subgroup 1 (*p* < 0.01). Interestingly, subgroup 2 also had significantly lower ratings of excess loudness variation than subgroup 1 (*p* < 0.01).

#### 3.3.2. Dimension 2

D2 was associated with hoarse voice (r = 0.422), overall loudness (r = −421), and variable rate (r = −414). Specifically, hoarse voice and variable rate were exclusively correlated with D2. Furthermore, D2 was the only dimension that was also significantly correlated with talkers’ sex [r (47) = −356, *p* = 0.01].

As shown in Table 3, Kruskal–Wallis H tests revealed significant between-group effects for hoarse voice, overall loudness, and variable rate (*p* < 0.05). The subsequent results of Dunn’s post-hoc test adjusted with Bonferroni correction (see Table 4 and Figure 6) for each variable that showed significant Kruskal–Wallis H test results and moderately correlated with D2 indicated that subgroup 3 had significantly higher ratings of variable rate than subgroup 4 (*p* = 0.04). Although not significant, subgroup 3 tended to have lower ratings of hoarse voice than subgroup 1 (*p* = 0.08), and subgroup 4 tended to have greater overall loudness ratings than subgroup 1 (*p* = 0.05). Finally, the Fisher’s exact test examining sex effects also did not reach significance (*p* = 0.07); however, subgroup 3 consisted of twelve females and one male, whereas all other subgroups were more balanced in their distribution of female and male talkers (see also Table 1).

#### 3.3.3. Dimension 3

Overall, D3 was moderately associated with perceptual speech characteristics related to articulation (imprecise consonants (r = 0.547), irregular articulatory breakdown (r = 0.487), vowels distorted (r = 0.486)), as well as perceptual ratings of strained-strangled voice (r = 0.539), intelligibility (r = 0.497), and reduced stress (r = 0.412). It should be noted that relative to D1, D3 had weaker correlations with all of the perceptual speech characteristics that were shared by D1 and D3 (e.g., imprecise consonants, vowels distorted, irregular articulatory breakdown, and intelligibility). In contrast to D1, however, D3 was moderately and exclusively correlated with the perceptual ratings of strained-strangled voice (r = 0.539) and reduced stress (r = 0.412).

As shown in Table 3, Kruskal–Wallis H tests revealed significant between-group effects for all of the perceptual speech characteristics that were moderately correlated with D3 (*p* < 0.05), except for reduced stress. Dunn’s post-hoc test (with adjusted *p*-values using Bonferroni corrections, see Table 4 and Figure 6) was applied for each variable that reached significant Kruskal–Wallis H test results, and was moderately correlated with D3 showed the following patterns: subgroup 4 had significantly lower ratings than subgroups 1 and 3 with regards to irregular articulatory breakdown and intelligibility (*p* < 0.05). Furthermore, perceptual ratings of vowels distorted were significantly lower for subgroup 4 than subgroup 1 (*p* < 0.01) and tended to be lower for subgroup 4 than subgroup 3 (*p* = 0.06). In addition, subgroup 4 had significantly lower ratings of imprecise consonants than subgroup 1 (*p* < 0.01). Finally, subgroup 1 had significantly greater ratings for strain-strangled voice than subgroups 2 and 4 (*p* < 0.05).

## 4. Discussion

The purpose of the current study was to compare an auditory free classification approach (FREECLASS) and a statistical clustering approach (STATCLUSTER) to determine to what extent these approaches overlap in their subgrouping decisions. Furthermore, the perceptual speech characteristics that contributed to the subgrouping decisions of each approach were compared. The chi-square analysis revealed significant overlap between the two approaches. Relative to the STATCLUSTER approach in which overall dysarthria severity (index by bizarreness ratings) and speech rate abnormalities differentiated dysarthria subgroups, the FREECLASS dysarthria subgroups were driven by abnormal voice quality (i.e., strained-strangled voice, hoarse voice) and the talker’s sex, in addition to dysarthria severity and speech rate, which had also been identified for the STATCLUSTER approach.

### 4.1. Overlap between the FREECLASS and the STATCLUSTER Approach

The findings of the FREECLASS approach revealed four unique dysarthria subgroups of talkers with HD, which is congruent with the number of subgroups identified using the STATCLUSTER approach by Diehl and colleagues (2019) [10] based on perceptual ratings from the Mayo Clinic dysarthria rating scale. However, not every listener grouped talkers with HD into four subgroups. The number of groups identified by listeners ranged from 4 to 13. Such variability in grouping decisions among listeners has been documented previously (see [6,17]) and suggests that grouping strategies employed during a FREECLASS approach and the saliency of perceptual speech characteristics that contribute to grouping decisions are highly subjective. Nevertheless, the convergence on four dysarthria subgroups among talkers with HD, regardless of the approach, solidifies the notion that dysarthria in HD can manifest in different ways, and this heterogeneity should be considered in the design of future studies on dysarthria in HD.

Overall, the overlap of subgroup membership ranged from 50% to 78.6%. Thus, for at least half of the talkers with HD who participated in our study, statistical grouping decisions based on ratings of perceptual speech characteristics were congruent with listeners’ similarity judgments of dysarthric speech patterns. Specifically, subgroup 4 of the current study using the FREECLASS approach consisted entirely of the talkers from subgroups 1 (fast rate/mild dysarthria) and subgroup 4 (normal rate/mild dysarthria) previously established using the STATCLUSTER approach. Furthermore, talkers of subgroup 2 of the FREECLASS approach belonged either to subgroup 2 (slow rate/moderate dysarthria) or subgroup 3 (slow rate/mild dysarthria) of the STATCLUSTER approach.

However, the two grouping approaches did not yield a complete overlap in subgroups, suggesting that listeners weighed perceptual speech characteristics during the auditory free classification task differently than the perceptual ratings of the Mayo Clinic dysarthria rating scale had indicated. That is, perceptual speech characteristics that were rated higher or lower in the Mayo system may influence grouping decisions to a lesser or greater extent during the auditory free classification task, respectively. For example, a perceptual speech feature may receive a lower rating, indicating mild impairment (e.g., strained-strangled voice), but may still be more salient to listeners than other perceptual speech features that were rated as more severely impaired (e.g., slow speech). Furthermore, although several perceptual speech characteristics may be rated equally deviant on the Mayo Clinic perceptual rating scale, one deviant perceptual speech characteristic may be more salient and may contribute to the listeners’ grouping decision to a greater extent than the others. The algorithm in the STATCLUSTER approach assumes linear associations between perceptual ratings and their saliency. However, as discussed below, the findings of the current study suggest that such associations may be non-linear, at least for some perceptual speech characteristics.

### 4.2. Perceptual Speech Characteristics Contributing to Subgrouping Decisions

The strong correlations of D1 with bizarreness ratings and ratings of perceived speech rate indicate that these two variables played a prominent role in the FREECLASS subgrouping decisions. However, additional perceptual speech characteristics also impacted the listeners’ grouping decisions during the auditory free classification task (i.e., variable rate, strained-strangled voice, hoarse voice, talker’s sex). In the following sections, the findings of each dimension will be discussed in more detail.

#### 4.2.1. Interpretations of D1

D1 findings suggest that grouping decisions were primarily based on overall bizarreness and intelligibility ratings, as well as ratings of several perceptual speech characteristics (i.e., perceived speech rate, excess and equal stress, prolonged intervals, inappropriate silences, imprecise consonants, prolonged phonemes, vowels distorted, irregular articulatory breakdown, monopitch, and excess loudness variation). These perceptual speech characteristics likely contributed to the overall bizarreness and intelligibility ratings. Based on our findings of MDS-related analyses (see Figure 5), D1 likely explains the perceptual speech characteristics that account for subgroup 4. Although several perceptual speech characteristics were identified in D1, many were moderate to strongly collinear with the overall bizarreness ratings (see Table 5). Thus, it is conceivable that listeners grouped talkers in subgroup 4 primarily because these talkers exhibited generally milder dysarthria symptoms than all other talkers, rather than because these talkers produced a specific combination of perceptual speech characteristics. However, we cannot exclude the possibility that a certain combination of perceptual speech characteristics associated with D1 accounted for the listeners’ grouping decision for subgroup 4. For example, pairwise between-group comparisons suggest that ratings of speech rate and monopitch differed between subgroup 4 and subgroup 2, whereas ratings for irregular articulatory breakdowns differentiated subgroup 4 and 1, as well as subgroup 4 and 3. Nevertheless, these perceptual speech characteristics were moderately correlated with the overall bizarreness ratings.

The overall bizarreness rating and perceived speech rate, which were two of the main contributors of the grouping decision in the FREECLASS approach, primarily accounted also for the dysarthria subgroups established by the STATCLUSTER approach in the study by Diehl and colleagues (2019) [10]. Given that both studies were based on the same cohort of talkers with HD, this finding is not surprising. However, it is important to note that speech impairment severity and speech rate were also two main variables that contributed to the listeners’ grouping decisions in the study by Lansford and colleagues (2014) [6]. In contrast to our study, their study included talkers with a variety of neurological conditions and dysarthria types. Based on the parallel findings, one could speculate that listeners may take a more holistic approach to differentiate talkers with dysarthria by focusing on the overall dysarthria severity first, and only considering specific speech perceptual characteristics (i.e., speech rate, voice quality) as a secondary strategy to further differentiate talkers within a specific dysarthria severity range, regardless of the talkers’ underlying etiology. Although our speech samples only included talkers with HD who exhibited mild dysarthria (70–100% intelligibility), the range in perceived bizarreness ratings across talkers still predominantly impacted the listeners’ decisions.

#### 4.2.2. Interpretations of D2

Findings of D2 suggest that grouping decisions were based on variable rate, hoarse voice quality, and talker’s sex. In contrast to overall bizarreness ratings accounting for most of the grouping decisions in D1, the findings of D2 suggest that specific speech perceptual characteristics contributed to grouping decisions in D2. Based on the MDS-related analyses (see Figure 5), the perceptual speech characteristics associated with D2 likely explain grouping decisions of subgroup 3, with only one specific perceptual speech characteristic differing talkers of subgroup 3 from those of subgroup 1 (i.e., variable rate) and 4 (i.e., perceived hoarse voice quality). Furthermore, the talker’s sex showed a trend as a potential grouping variable differentiating subgroup 3 from subgroup 2. The finding that the talker’s sex may contribute to the listeners’ grouping decision aligns with previous findings by Clopper and colleagues (2004) [17]. In their study, the perceived sex of the talker was identified as an important perceptual speech characteristic in the similarity judgments of regional dialects.

#### 4.2.3. Interpretations of D3

Findings of D3 suggest that specific grouping decisions were primarily based on strained-strangled voice quality, a characteristic that was exclusively correlated with D3. Based on the MDS-related analyses (see Figure 5), the perceptual speech characteristics of D3 likely explain the listeners’ strategy in distinguishing the talkers in subgroup 1 from the talkers in subgroup 2. Although the perceptual speech characteristics that describe articulatory performance deficits (i.e., imprecise consonants, irregular articulatory breakdown, and vowels distorted) and general speech impairment severity (i.e., speech intelligibility) were also moderately correlated with D3, none of the between-group comparisons of these perceptual speech characteristics reflected the listeners’ decisions in differentiating subgroup 1 from subgroup 2. However, the between-group comparisons of strained-strangled voice demonstrated that the ratings of strained-strangled voice quality were significantly greater in subgroup 1 than in subgroups 2 and 4. That is, talkers in subgroup 1 were rated to produce a more pronounced strained-strangled voice quality than talkers in subgroups 2 and 4, who typically did not exhibit a strained-strangled voice or only a mild impairment. This finding is also consistent with the previous finding by Lansford and colleagues (2014) [6], who also suggested that strained-strangled voice quality can account, in part, for grouping decisions. Therefore, strained-strangled voice quality appears perceptually more salient to listeners than some of the other perceptual speech characteristics that received similar ratings but did not contribute to grouping decisions.

Finally, it is also important to mention the one talker who was excluded from further analysis because the talker did not fit with any of the four identified subgroups of the additive similarity tree cluster analysis. This talker presented with a fast speaking rate, monopitch, imprecise consonants, and irregular articulatory breakdown. Although the excluded talker was perceived to have a fast speaking rate like the talkers in subgroup 4, the perceptual speech characteristics of the excluded talker were shared across those of the other three subgroups. Therefore, it is possible that the excluded talker represents another dysarthria subgroup of HD. Future research with a new, larger sample of talkers with HD is warranted to replicate and extend the current findings.

### 4.3. Limitations

Because listeners were instructed to group talkers based on similarity during an auditory free classification task, the resulting grouping decisions may be subjected to response bias that forces listeners to form more than one group. However, due to the nature of the auditory free classification task, it is impossible to remove such a response bias. Nevertheless, previous studies have used a bias parameter to determine the listeners’ response biases. These studies have shown that an auditory free classification task significantly reduces response bias compared to a forced-choice task providing predetermined perceptual features [26,27]. However, it is possible that listeners were biased or primed in their grouping decisions due to unintended focus on deviant perceptual characteristics as a result of their familiarity and training in motor speech disorders. Without such influence, they may have also focused on additional perceptual speech features, such as the talkers’ age. In addition, the reliability of listeners’ grouping decisions should also be tested in future studies. So far, such reliability testing has not been conducted; however, based on findings of a wide range of responses, such an analysis is warranted to determine trial-to-trial variability within listeners. Finally, it is possible that the perceived gender of talkers could have contributed to the listeners’ grouping decision. However, we did not ask the listeners about their perception of the talkers’ gender, and therefore we could not consider this possibility in the current study. 

## 5. Conclusions

The current study revealed substantial overlap between subgroups of talkers with HD based on the FREECLASS approach and the subgroups based on the STATCLUSTER approach using the perceptual ratings of the Mayo Clinic dysarthria rating scale. This finding suggests that talkers with dysarthria due to HD could be differentiated based on severity (early stage dysarthria, progressed stage of dysarthria), as well as based on their speech rate characteristics (i.e., slow, adequate, fast) and vocal quality abnormalities (i.e., hoarse voice, adequate voice, strained-strangled voice). However, the lack of complete overlap across the two grouping approaches further indicates that listeners may find specific perceptual speech characteristics more salient than suggested by ratings of Mayo Clinic perceptual dimensions.

As a final note, it should be pointed out that the aim of this paper was not to identify a variety of potentially mixed dysarthria types for talkers with HD. Rather, our findings suggest that specific deviant perceptual speech characteristics should be considered when there is a need to minimize the heterogeneity of dysarthria symptoms in talkers with HD. Specifically, the consideration of rate- and voice-related perceptual speech characteristics, in addition to general severity ratings, can serve as a grouping strategy or as inclusion/exclusion criteria for research participants with HD. Such refinements in the study design may optimize investigations on underlying pathomechanisms of dysarthria in HD, as well as on the effectiveness of pharmaceutical and behavioral treatment approaches within specific dysarthria subgroups of HD.

## Figures and Tables

**Table 1:** Number of talkers overlapping between the identified four subgroups from the FREECLASS approach and the subgroups identified previously using computer-generated cluster analysis based on a dysarthria rating scale (STATCLUSTER) [10].

Identified Subgroups	Subgroups	# of Overlapped
(# of Talkers)	(Diehl et al., 2019 [10])	Talkers (%)
Subgroup 1	Subgroup 1 (n = 9)	2 (20)
(10 talkers; M = 3, F = 7)	Subgroup 2 (n = 9)	**5** (**50**)
	Subgroup 3 (n = 16)	2 (20)
	Subgroup 4 (n = 14)	1 (10)
Subgroup 2	Subgroup 2 (n = 9)	3 (30)
(10 talkers; M = 6, F = 4)	Subgroup 3 (n = 16)	**7** (**70**)
Subgroup 3	Subgroup 1 (n = 9)	3 (23.1)
(13 talkers; M = 1, F = 12)	Subgroup 2 (n = 9)	1 (7.7)
	Subgroup 3 (n = 16)	**7** (**53.8**)
	Subgroup 4 (n = 14)	2 (15.4)
Subgroup 4	Subgroup 1 (n = 9)	3 (21.4)
(14 talkers; M = 5, F = 9)	Subgroup 4 (n = 14)	**11** (**78.6**)

M, Males; F, Females.

**Table 2:** Significant findings of the correlation analyses between MDS dimensions and 32 speech characteristics with the addition of sex.

Dimension 1 Variable (Category)	r	Dimension 2 Variable (Category)	r	Dimension 3 Variable (Category)	r
Bizarreness ^a b^ (GI)	0.835	Hoarse voice ^a b^ (VQ)	0.422	Imprecise consonants ^a b^ (A)	0.547
Rate ^a b^ (P)	−0.710	Loudness overall ^a b^ (L)	−0.421	Strained-strangled voice ^a b^ (VQ)	0.539
Excess and equal stress ^a b^ (P)	0.616	Variable rate ^a b^ (P)	−0.414	Intelligibility ^a b^ (GI)	0.497
Intervals prolonged ^a b^ (P)	0.609	Breathy voice (VQ)	0.398	Irregular articulatory breakdown ^a b^ (A)	0.487
Inappropriate silences ^a b^ (P)	0.597	Sex	−0.356	Vowels distorted ^a b^ (A)	0.486
Imprecise consonants ^a b^ (A)	0.583	Reduced stress (P)	0.333	Reduced stress ^a^ (P)	0.412
Phonemes prolonged ^a b^ (A)	0.572	Harsh voice ^b^ (VQ)	0.302	Voice stoppages (P)	0.382
Intelligibility ^a b^ (GI)	0.550			Voice tremor (PC)	0.369
Vowels distorted ^a b^ (A)	0.536			Monoloudness ^b^ (L)	−0.349
Irregular articulatory breakdown ^a b^ (A)	0.521			Rate ^b^ (P)	0.348
Monopitch ^a b^ (PC)	0.460			Excess and equal stress ^b^ (P)	−0.334
Excess loudness variation ^a b^ (L)	0.414				
Alternating loudness ^a^ (L)	0.410				
Monoloudness ^b^ (L)	0.394				
Loudness overall ^b^ (L)	−0.389				
Harsh voice ^b^ (VQ)	0.327				
Loudness decay (L)	0.308				

Note: All speech characteristics with significant correlations (*p* < 0.05) ranked in order from the highest to lowest correlations under each dimension. A, articulation; GI, general impression; L, loudness; P, prosody; PC, pitch characteristics; R, respiration; VQ, voice quality; ^a^ Characteristics that have correlation coefficients 0.4 or greater; ^b^ Characteristics that have significant subgroup effects (*p* < 0.05) from either the Kruskal–Wallis test or Fisher’s exact test.

**Table 3:** The results of Kruskal–Wallis H test on variables that are significantly correlated with the revealed three dimensions from MDS analysis.

Variable	Kruskal–Wallis Test H	df	Sig.
Alternating loudness	7.02	3	0.071
Bizarreness	27.58	3	<0.001 *
Breathy voice	3.27	3	0.352
Excess and equal stress	17.83	3	<0.001 *
Excess loudness variation	16.89	3	0.001 *
Harsh voice	8.64	3	0.034 *
Hoarse voice	8.23	3	0.042 *
Imprecise consonants	17.1	3	0.001 *
Inappropriate silences	19.47	3	<0.001 *
Intelligibility	21.11	3	<0.001 *
Intervals prolonged	14.46	3	0.002 *
Irregular articulatory breakdown	16.23	3	0.001 *
Loudness decay	2.59	3	0.458
Loudness overall	8.59	3	0.035 *
Monoloudness	14.53	3	0.002 *
Monopitch	10.72	3	0.013 *
Phonemes prolonged	15.28	3	0.002 *
Rate	21.39	3	<0.001 *
Reduced stress	7.56	3	0.056
Sex	NA	NA	NA
Strained-strangled voice	11.62	3	0.009 *
Variable rate	9.12	3	0.028 *
Voice stoppages	4.32	3	0.229
Voice tremor	7.29	3	0.063
Vowels distorted	15.25	3	0.002 *

* *p* < 0.05; NA, not adequate for the specific analysis.

**Table 4:** Multiple comparisons for each variable that showed significant Kruskal–Wallis H test results and moderate correlations with the dimensions from MDS analysis.

Variable (Correlated Dimensions) Subgroups	*z*	*p*	Variable (Correlated Dimensions) Subgroups	*z*	*p*
Bizarreness			Excess and equal stress		
(D1)			(D1)		
4-3	18.5	0.003 *	4-3	17.4	0.006 *
4-2	19.9	0.003 *	4-2	20.9	0.001 *
4-1	27.8	<0.001 *	4-1	16.1	0.027 *
Excess loudness			Inappropriate silences		
variation			(D1)		
(D1)			4-3	19.7	0.001 *
4-1	18.0	0.001 *	4-2	20.7	0.002 *
2-1	16.5	0.007 *	4-1	16.2	0.025 *
Intervals prolonged			Rate		
(D1)			(D1)		
4-2	19.7	0.003 *	4-2	−25.9	<0.001 *
4-1	15.7	0.031 *	4-1	−14.5	0.064
			3-2	−14.9	0.059
Monopitch			Phonemes prolonged		
(D1)			(D1)		
4-2	18.2	0.008 *	4-2	15.1	0.045 *
			4-1	20.8	0.001 *
Intelligibility			Imprecise Consonants		
(D1 and D3)			(D1 and D3)		
4-3	14.1	0.040 *	4-1	23.2	<0.001 *
4-1	25.4	<0.001 *			
Irregular articulatory breakdown			Vowels distorted		
(D1 and D3)			(D1 and D3)		
4-3	15.2	0.024 *	4-3	13.3	0.068
4-1	20.7	0.002 *	4-1	21.3	0.001 *
Hoarse Voice			Variable rate		
(D2)			(D2)		
3-1	11.4	0.084	4-3	14.2	0.04 *
Loudness overall			Strained-strangled voice		
(D2)			(D3)		
4-1	−13.7	0.052	4-1	16.7	0.017 *
			2-1	7.3	0.041 *

* *p* < 0.05 (significance values have been adjusted by the Bonferroni correction for multiple tests).

**Table 5:** Ranked correlations between the ratings of overall bizarreness and the ratings of all the perceptual speech characteristics that were moderately correlated with the identified dimensions.

Variable	Pearson Correlation	Sig.	Correlated Dimensions
Intelligibility	0.789	<0.001 *	1 and 3
Imprecise consonants	0.764	<0.001 *	1 and 3
Irregular articulatory breakdown	0.730	<0.001 *	1 and 3
Vowels distorted	0.664	<0.001 *	1 and 3
Inappropriate silences	0.619	<0.001 *	1
Alternating loudness	0.520	<0.001 *	1
Intervals prolonged	0.515	<0.001 *	1
Excess and equal stress	0.494	<0.001 *	1 and 3
Monopitch	0.487	0.001 *	1
Rate	−0.474	0.001 *	1 and 3
Reduced stress	0.449	0.002 *	2 and 3
Excess loudness variation	0.437	0.002 *	1
Harsh voice	0.423	0.003 *	1 and 2
Phonemes prolonged	0.416	0.004 *	1
Strained-strangled voice	0.415	0.004 *	3
Loudness overall	−0.412	0.004 *	1 and 2
Breathy voice	0.391	0.007 *	2
Monoloudness	0.385	0.007 *	1 and 3
Voice stoppages	0.380	0.008 *	3
Variable rate	0.368	<0.001 *	2
Hoarse voice	0.284	0.053	2
Voice tremor	0.225	0.128	3

* *p* < 0.05; all speech characteristics that had significant correlations with the identified dimensions are ranked in order from the highest to lowest correlations with the ratings of overall bizarreness.

## Data Availability

Data that support the findings of this study are available from the corresponding author upon reasonable request.
